# A Meteorological Table, by Dr. Higgins, of Brompton. From February 22, to March 22, 1805

**Published:** 1805-04-01

**Authors:** 


					A Meteorological Table, by Dr. Higgins, of Brampton.
From February 22, to March 22, 1805.
LiX.
2 Z
2-3
24
25
26
27
28
h 1
2
3
4
5
6
7
8
9
10
11
12
13
14
if
16
17
18
19
20
21
22
Height or the Baro-
meter. Inches.
U ?
30.00 30,
?34|
?33
.10!
.121
.01
29*79'29
.69)
30.2830
.62
.50
.26
.38
?77
.56
.12
29-92
30.27
?35
?4?|
.09
?03
?3i
?19]
.22
.63
.58
.56
. -36|
00!
47
II
13
HI
oo|
72
84I
?37
.64)
?4 5!
.28
.4?
?79|
.50]
.02
3.92
>-34|
?35
?37|
.01
.11
.36
?13
.28
.62
.56
?49
?32
3 "
u-Si
?2
30.06
?53!
.01
?13
?I5,
29.80
.65
?96
30-51
.60!
?33
.36
.48
?71
.28
29.94
30.06
?35
.38
.29
29.92
30.26
?25
.06
?46
.65
.56
.42
?32
WEATHER.
Cloudy.
Fair, rain in the night.
Rain, fair in the evening.
Fair.
Showery. 1
Cloudy, rain in the evening.
Rain and hail fhowers
Windy, hail lhowers, & fnow in night.
Cloudy, with light thowers
Cloudy, rain in the night.
Cloudy with wind.
Fair.
Cloudy. *?
Fair.
Fair.
Cloudy.
Fair.
Fair.
Fair.
Fair.
Fair, cloudy in the evening.
Cloudy.
Fair.
Fair.
Small ?
Fair, . 1 in the night.
Rain.
Cloudy.
Windy.

				

